# Consumer Segmentation and Gender-Specific Adoption Pathways for Edible Insects: Evidence from Saudi Arabia

**DOI:** 10.3390/insects17070688

**Published:** 2026-07-02

**Authors:** Hala Hazam Al-Otaibi, Samar Refat Alabdulmohsen

**Affiliations:** Department of Food and Nutrition Science, College of Agricultural and Food Sciences, King Faisal University, Al-Hofuf 31982, Saudi Arabia

**Keywords:** consumer segmentation, edible insects, entomophagy acceptance, gender differences, Saudi Arabia, behavioral determinants, sustainable diets

## Abstract

Saudi Arabia relies heavily on food imports, making sustainable protein integration a national priority under Vision 2030. However, large-scale adoption of edible insects remains limited by psychological, cultural, and religious barriers that vary across consumer groups. This study develops a behavioral segmentation framework using a cross-sectional survey of 2208 Saudi adults, representing one of the largest behavioral studies of edible insect adoption in the Saudi context. Three distinct segments emerged from hierarchical cluster analysis: Resistant Rejectors (43.1%), Conditional Adopters (33.0%), and Early Adopters (23.9%). Gender-stratified ordinal logistic regression revealed distinct adoption pathways: among men, willingness was primarily associated with interest in entomophagy (OR = 3.46) and social influence (OR = 2.51); among women, prior consumption experience was the strongest facilitator (OR = 3.07), while religious concern represented the most substantial barrier (OR = 0.15). Acceptance of insects as animal feed was consistently higher than direct consumption across all segments, supporting a three-stage integration model (feed → processed foods → direct consumption) as a culturally pragmatic transition pathway. These findings provide actionable, segment-specific insights for policymakers, industry stakeholders, and food system innovators.

## Simple Summary

Saudi Arabia relies heavily on food imports, making the search for sustainable, locally produced protein sources an important national priority. Edible insects such as grasshoppers and locusts are nutritious, environmentally efficient, and require far less land and water than conventional meat. However, many people are hesitant to eat insects due to cultural norms, religious concerns, and feelings of disgust. This study surveyed 2208 adults across Saudi Arabia to understand who is willing to consume insects and why. Three groups were identified: those who refuse, those who may accept under certain conditions, and those already open to trying. Men and women showed different patterns. Men were more influenced by environmental concerns and social influence, while women were more influenced by personal experience and clarity about whether insects are permissible to eat. Importantly, even those unwilling to eat insects directly were more accepting of meat and fish raised on insect-based feed. These findings suggest a gradual approach, starting with animal feed, then using insects in familiar foods, and finally introducing them as visible products. This approach may help support more sustainable food systems.

## 1. Introduction

The global transition toward sustainable food systems has intensified the search for alternative protein sources capable of meeting rising demand while minimizing environmental impact. The edible insect market has emerged as a notable growth sector in this context; the global edible insects’ market was estimated at USD 1.35 billion in 2024 and is projected to reach USD 4.38 billion by 2030, growing at a compound annual growth rate (CAGR) of 25.1% [1]. This trajectory is underpinned by significant environmental advantages; insect farming emits up to 100 times fewer greenhouse gases than conventional livestock production and requires 50–90% less land and water, closely aligning with global circular bioeconomy goals [2]. This growth is largely attributed to escalating demand for sustainable proteins, with conventional livestock production estimated to account for nearly 14.5% of anthropogenic greenhouse gas emissions globally [3]. In Saudi Arabia, food self-sufficiency remains a strategic priority under Vision 2030, particularly given that the Kingdom currently imports over 80% of its food, with animal protein imports valued at over USD 4 billion annually [4]. Edible insects have gained increasing attention due to their high nutritional value, efficient feed conversion, and relatively lower environmental footprint compared to conventional livestock [5,6]. Despite these advantages, large-scale adoption remains limited, particularly in culturally sensitive regions where dietary practices are deeply shaped by social norms, religious interpretations, and psychological perceptions.

Existing research on entomophagy has primarily focused on measuring consumer acceptance and identifying key determinants such as disgust sensitivity, familiarity, and perceived health risks [7,8]. While these studies provide valuable insights, they often treat populations as relatively homogeneous groups, overlooking the heterogeneity of consumer behavior and the existence of distinct adoption pathways. This limitation may restrict the ability to translate behavioral findings into practical strategies for market development and food system integration.

In recent years, scholars have emphasized the importance of moving beyond descriptive acceptance models toward more dynamic frameworks that account for behavioral diversity and decision-making processes [9,10]. Segmentation-based approaches may offer a promising avenue for identifying consumer subgroups with different motivations, barriers, and levels of readiness for adoption, enabling more targeted and scalable intervention strategies. Such approaches are especially relevant in contexts where cultural and religious factors may play a critical role in shaping food choices.

Saudi Arabia presents a unique case for examining these dynamics. Although certain forms of insect consumption, such as locusts, have historical and religious acceptance within Islamic dietary traditions, modern dietary patterns appear to reflect increasing resistance toward entomophagy. This apparent contradiction creates a “cultural transition gap,” where traditional knowledge coexists with contemporary rejection. Understanding this gap may be essential for designing effective strategies to introduce sustainable protein alternatives.

Despite growing interest in entomophagy, research that explicitly examines how acceptance differs between men and women within Muslim-majority and Gulf populations remains scarce; existing Saudi and regional studies have largely reported aggregate acceptance without disentangling gender conditioned barriers and facilitators. This constitutes a clear knowledge gap, because gender appears to interact with religious and safety related concerns in ways that generic, population level acceptance models cannot capture.

Theoretically, this study is informed by three complementary perspectives. First, the Theory of Planned Behavior conceptualizes willingness to consume insects as being influenced by attitudes, subjective norms (operationalized here as social influence), and perceived behavioral control [11]. Second, food neophobia, the reluctance to eat unfamiliar foods [12], and the psychology of disgust [13] help explain the strong rejection responses commonly observed toward whole insects. Third, gender schema and household role perspectives suggest that women, who often play a central role in household food selection and dietary decision-making in many cultural settings, may weigh permissibility and safety concerns more heavily than men. Together, these frameworks motivate the segmentation and gender-stratified approach adopted in this study.

This study contributes to the existing literature in four keyways. First, it represents to the best of our knowledge the largest behavioral study (*n* = 2208) on edible insect adoption conducted in any Muslim-majority country, providing statistical power for robust subgroup analyses that were not available in prior smaller studies. Second, while previous segmentation studies in Western contexts have identified consumer profiles based primarily on disgust, neophobia, and environmental concern, this study explicitly integrates religious-cultural dimensions and gender-conditioned pathways into the segmentation framework. Third, it empirically examines the gateway hypothesis [14] by demonstrating that even resistant segments show significantly higher acceptance of indirect consumption (insect-based animal feed) than direct consumption, providing actionable evidence for staged market entry strategies. Fourth, it addresses the gap between consumer behavior research and food system development by translating behavioral segments into a structured, three-stage integration model with concrete product, messaging, and policy implications relevant to Saudi Vision 2030 and broader Gulf Cooperation Council (GCC) contexts. From an applied standpoint, identifying distinct consumer segments and gender-specific pathways enables the design of targeted marketing and communication strategies rather than one size fits all messaging thereby supporting the promotion of sustainable, locally produced protein in line with Saudi Vision 2030.

## 2. Materials and Methods

### 2.1. Study Design and Participants

A cross-sectional online survey was conducted between October 2024 and January 2025 among Saudi adults aged 18 years and above. Eligibility was limited to Saudi nationals aged 18 years or older. No additional exclusion criteria were applied; participants with more than 20% missing data were removed during data cleaning. In addition, responses were screened for quality, and cases exhibiting implausible or inconsistent response patterns (e.g., straight lining or contradictory answers) were excluded where applicable. For the remaining dataset, missing values were minimal (<5% per variable), and analyses were conducted using listwise deletion. An a priori power analysis using G*Power 3.1 indicated that a minimum sample of 1067 participants would be required to detect small-to-medium effects (f^2^ = 0.02) in ordinal logistic regression with 12 predictors at α = 0.05 and statistical power of 0.95. To allow for sufficient statistical power in subgroup analyses (gender × segment interactions), recruitment continued until more than 2000 valid responses were obtained. Although no formal power formula exists for cluster analysis, a minimum of 200–300 participants per anticipated segment is generally considered adequate for stable cluster solutions [15]; with the smallest cluster in this study comprising 528 participants, the achieved sample size is substantially above this threshold.

Participants were recruited via convenience and snowball sampling through social media platforms (Telegram, X, WhatsApp). A total of 2612 individuals accessed the survey link; of these, 2396 began the questionnaire, and 2208 completed all sections, with 188 responses excluded due to more than 20% missing data (final completion rate: 92.2% of those who started; 84.5% of those who accessed the link). All participants provided digital informed consent. Participation was voluntary, and respondents could withdraw at any time without consequence. The survey was anonymous and required approximately 15 min to complete. To minimize duplicate responses, participation was restricted to one response per device/IP address where feasible. The study was conducted in accordance with the Declaration of Helsinki and received ethical approval from King Faisal University (KFU-REC-2024-OCT-ETHICS2710).

### 2.2. Measures

Data collection comprised a structured questionnaire designed to capture sociodemographic characteristics, behavioral determinants, and attitudinal constructs related to edible insect consumption. Sociodemographic variables included age, gender, educational level, monthly income, employment status, and region of residence. Behavioral exposure was assessed through measures of prior experience with insect-based foods and familiarity with insect consumption, defined as knowing someone who has consumed insects (e.g., locusts) [9].

Social influence was evaluated using a single-item measure (“I think I would be willing to try edible insects if I see everyone eating them”) [16]. Religious perception was assessed through a dichotomous belief statement (“It is forbidden (not halal) and should not be consumed”) [16,17,18].

Environmental concern was measured using a two-item scale (α = 0.78) [8], while health risk perception was assessed using three items addressing concerns related to food safety, including contamination, allergens, and microbial risks (α = 0.81) indicating acceptable internal consistency [18].

Attitudes toward edible insects were measured using the validated Entomophagy Attitude Questionnaire (EAQ) [19], which includes three subscales: Disgust (5 items, α = 0.941), Interest (3 items, α = 0.878), and Feeding Animals (2 items, α = 0.742). All EAQ items were rated on a seven-point Likert scale ranging from 1 (strongly disagree) to 7 (strongly agree), where higher Disgust scores indicate more negative attitudes toward direct entomophagy, higher Interest scores indicate more positive attitudes, and higher Feeding Animals scores indicate more positive attitudes toward indirect entomophagy through animal feed applications. Prior to analysis, confirmatory factor analysis (CFA) was conducted to assess the factor structure of the Entomophagy Attitude Questionnaire (EAQ) in the study sample. The results supported the expected three-factor structure, indicating acceptable construct validity for subsequent analyses.

Willingness to consume edible insects was assessed using a four-item scale (α = 0.84), adapted from Liu et al. [20], Ordonez Lopez et al. [21], and Szlachciuk and Żakowska-Biemans [18]. Responses were rated on a five-point Likert scale and summed (range: 4–20). Following the cut-points established by the original scale developers, scores were categorized into three ordinal levels: unwilling (≤4, indicating consistent strong disagreement across all items), uncertain (5–11), and willing (≥12). This categorization was consistent with prior studies using this instrument and enabled ordinal logistic regression modeling. Categorization was applied to enhance interpretability and ensure compatibility with ordinal regression assumptions, while maintaining consistency with prior validation studies. Although single-item measures limit reliability, they are widely used in exploratory consumer research where constructs are concrete and context-specific (e.g., perceived religious permissibility) and were considered appropriate to reduce respondent burden in a multi-construct survey [9,18].

To ensure cultural and linguistic appropriateness, the survey instrument was translated using a forward-backward translation protocol. A pilot study was conducted with 20 Saudi adults (10 men and 10 women) to evaluate clarity and reliability. Minor wording adjustments were made following pilot testing to improve clarity and cultural appropriateness; no structural changes to the scales were required. All scales demonstrated acceptable internal consistency (Cronbach’s α > 0.75). It should be noted that social influence, religious perception, and familiarity were each assessed using single-item measures; while this approach is common in exploratory consumer research and is consistent with prior entomophagy studies [9,22], it may limit psychometric robustness and is acknowledged as a study limitation.

### 2.3. Statistical Analysis

Behavioral segmentation was performed using hierarchical clustering followed by k-means refinement procedure on six standardized (*z*-score) variables: disgust sensitivity (EAQ-D), interest (EAQ-I), environmental concern, prior experience, social influence, and perceived religious constraints. These six variables were selected because they represent the primary theoretical constructs in the entomophagy acceptance literature and demonstrated the strongest bivariate associations with the outcome variable in preliminary analyses. To avoid redundancy and potential multicollinearity, only variables demonstrating both theoretical relevance and independent contribution in preliminary analyses were retained. Health risk perception was not included in the clustering model to preserve model parsimony and ensure clear differentiation between segments. Although theoretically relevant, it was excluded due to its conceptual proximity to disgust sensitivity, as both capture contamination- and safety-related concerns. Including both variables could reduce cluster interpretability and introduce redundancy. Therefore, health risk perception was examined separately in the regression analysis to assess its independent predictive effect on willingness. First, hierarchical cluster analysis using Ward’s linkage method with squared Euclidean distance was conducted to determine the optimal number of clusters. The agglomeration coefficient and dendrogram inspection suggested a three-cluster solution. The average silhouette coefficient of 0.42 indicates moderate cluster separation that is considered acceptable in applied behavioral research contexts.

To support this decision quantitatively, three internal validation indices were computed across solutions ranging from 2 to 6 clusters: the Calinski-Harabasz pseudo-F statistic (peaked at k = 3), the Davies-Bouldin index (minimized at k = 3), and the average silhouette coefficient (k = 3: 0.42; k = 2: 0.38; k = 4: 0.39). All three indices converged on k = 3 as the optimal solution. The average silhouette coefficient of 0.42 indicates reasonable cluster cohesion and separation; while this value falls in the moderate range by conventional benchmarks, it is consistent with silhouette values reported in consumer behavior segmentation studies in the food choice domain [22,23].

Following hierarchical clustering, k-means clustering with the three-cluster initial centroids was applied to refine cluster membership. Cluster stability was assessed through 100 bootstrap resamples; mean Jaccard similarity coefficients for the three clusters were 0.81, 0.78, and 0.76 respectively, all exceeding the 0.75 threshold recommended for stable cluster solutions. To externally validate the segmentation, mean differences across clusters on the primary outcome variable (willingness to consume edible insects, analyzed as a continuous summed score) were tested using one-way analysis of variance ANOVA with Bonferroni post hoc corrections, confirming that all three segments differed significantly from each other (*p* < 0.001). Segment membership was not included as a covariate in the ordinal logistic regression models; the regression analyses served an independent analytical purpose of identifying within-gender predictors of willingness across the full gender-stratified samples, rather than profiling cross-gender behavioral clusters.

**Figure 1:**
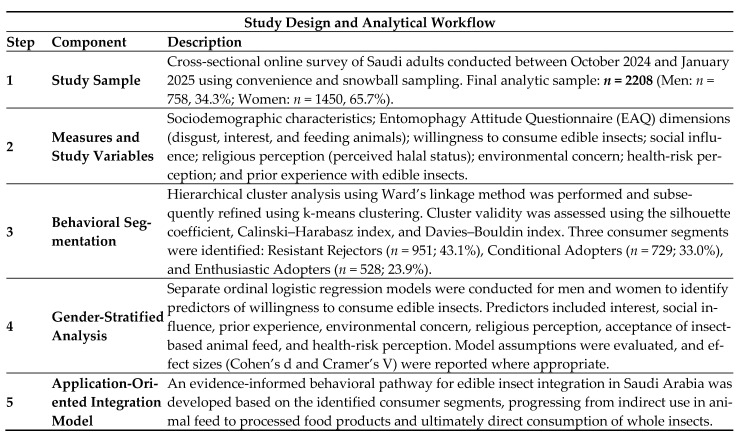
Overview of the study design and analytical workflow, from participant recruitment and measurement through behavioral segmentation and gender-stratified analyses to the development of the application-oriented integration model.

Ordinal logistic regression was used to identify key predictors of willingness to consume edible insects, stratified by gender. The proportional odds assumption was confirmed using the Test of Parallel Lines for both men (χ^2^ = 23.45, *p* = 0.102) and women (χ^2^ = 31.78, *p* = 0.068). Multicollinearity was assessed using variance inflation factors (VIF), all of which were below 3.0, indicating no significant collinearity concerns. Model fit was further evaluated using likelihood ratio tests and Nagelkerke R^2^ values. For gender comparisons, effect sizes were reported as Cramér’s V for chi-square tests and Cohen’s d for independent-samples *t*-tests. Confirmatory factor analysis (CFA) was conducted as part of the measurement validation process. All other statistical analyses, including hierarchical clustering, k-means refinement, ordinal logistic regression, and group comparisons, were performed using SPSS Statistics (version 25.0). Statistical significance was set at *p* < 0.05. Figure 1 illustrates the overall study design and analytical workflow used in the present study.

## 3. Results

### 3.1. Participant Characteristics

A total of 2208 Saudi adults participated, with 758 men (34.3%) and 1450 women (65.7%). The gender imbalance observed in the sample is consistent with typical online survey response patterns in the region and is partially addressed through gender-stratified analyses throughout. The sample was predominantly young, with the largest proportion aged 18–34 years. A significantly higher percentage of women (44.8%) than men (38.1%) were in this youngest age group (χ^2^ = 27.45, *p* < 0.001, Cramér’s V = 0.11, indicating a small effect size). No significant differences were observed between genders for income, education, or employment status (all *p* > 0.05, Cramér’s V < 0.05). Participants were recruited from all major regions of Saudi Arabia, with the highest representation from the central (32.9%) and eastern (31.7%) regions (Table 1).

### 3.2. Identification of Behavioral Segments

The hierarchical clustering followed by a k-means refinement procedure identified three distinct behavioral segments within the sample (*n* = 2208). Cluster 1, labeled ‘Resistant Rejectors,’ included 951 participants (43.1%); Cluster 2, ‘Conditional Adopters,’ included 729 participants (33.0%); and Cluster 3, ‘Early Adopters,’ included 528 participants (23.9%). The three-cluster solution achieved an average silhouette coefficient of 0.42, indicating moderate cluster separation that is considered acceptable in applied behavioral research contexts. Cluster centroids indicate distinct patterns across key variables. Table 2 presents the standardized cluster centroids (z-scores) for the three segments.

Segment 1: Resistant Rejectors (43.1% of the sample) is characterized by high disgust sensitivity, strong perceived health and religious concerns (>60% believe insects are not halal), low prior exposure to insect-based foods (<10% have tried), and minimal responsiveness to social influence (<15% influenced by peers). This segment included a higher proportion of women compared to men.
Segment 2: Conditional Adopters (33.0% of the sample) is characterized by moderate disgust and uncertainty, high responsiveness to product format (processed vs. whole insects), sensitivity to social influence and informational framing, and limited but not absent prior exposure (10–30% have tried). This segment showed moderate levels of willingness, which varied depending on product presentation.
Segment 3: Early Adopters (23.9% of the sample) is characterized by high interest in entomophagy, strong environmental motivation, lower disgust sensitivity, and higher likelihood of prior experience (>30% have tried). This segment includes a higher proportion of men.

### 3.3. Gender-Specific Pathways Across Segments

Segment membership revealed distinct gender-based behavioral patterns. Men were more frequently represented in the Early Adopters segment, driven by curiosity, environmental concern, and social influence. Women were more concentrated in the Resistant Rejectors segment, with adoption more strongly associated with prior experience, perceived safety, and religious interpretation. Table 3 shows gender differences in behavioral determinants across segments. All gender differences were significant at *p* < 0.001 with medium-to-large effect sizes (Cohen’s d ranging from 0.61 to 2.01 for continuous variables; Cramér’s V ranging from 0.17 to 0.35 for categorical variables), except familiarity (*p* = 0.293, Cramér’s V = 0.02).

### 3.4. Transitional Role of Indirect Consumption

These findings suggest that indirect consumption may function as a culturally pragmatic transitional bridge across all consumer groups. Notably, even within the Resistant Rejectors segment—the group most opposed to direct consumption—the majority of participants (men: 61.3%; women: 54.8%) scored above the EAQ-F midpoint (>3.5 on the 1–7 scale), indicating that animal feed represents a genuinely accessible entry point even for the most resistant consumers. Table 4 shows feed acceptance (EAQ-F) vs. direct willingness by segment and gender. Note that EAQ-F is measured on a 1–7 scale, while direct willingness is measured on a 1–5 scale; comparisons are therefore descriptive rather than directly numerical, and the consistent directional pattern (feed acceptance generally exceeding direct willingness within segments) is the key finding.

### 3.5. Predictors of Willingness to Consume: Gender-Stratified Ordinal Logistic Regression

Two ordinal logistic regression models were fitted separately for men (*n* = 758) and women (*n* = 1450) across the full sample. These models complement the segmentation analysis by identifying which psychological, cultural, and experiential variables most strongly predict willingness within each gender group, providing predictor-level evidence underlying the segment profiles described in Section 3.2.

For men, the model explained a large proportion of variance (Nagelkerke R^2^ = 0.68). The strongest positive associations were observed for interest in entomophagy (odds ratio, OR = 3.46, 95% confidence interval, CI [2.81, 4.26], *p* < 0.001), social influence (OR = 2.51, 95% CI [1.92, 3.28], *p* < 0.001), prior consumption experience (OR = 1.95, 95% CI [1.44, 2.64], *p* < 0.001), environmental concern (OR = 1.57, 95% CI [1.21, 2.04], *p* = 0.001), and acceptance of insects as animal feed (OR = 1.39, 95% CI [1.14, 1.70], *p* < 0.001). Variables negatively associated with willingness included disgust sensitivity (OR = 0.41, 95% CI [0.32, 0.53], *p* < 0.001), perceived non-halal status of insects (OR = 0.32, 95% CI [0.21, 0.49], *p* < 0.001), and health risk perception (OR = 0.73, 95% CI [0.58, 0.92], *p* = 0.007).

For women, the model explained a large proportion of variance (Nagelkerke R^2^ = 0.72). The strongest positive associations were observed for prior consumption experience (OR = 3.07, 95% CI [2.34, 4.03], *p* < 0.001), interest in entomophagy (OR = 1.76, 95% CI [1.38, 2.24], *p* < 0.001), familiarity with insect consumption (OR = 1.57, 95% CI [1.19, 2.07], *p* = 0.001), acceptance of insects as animal feed (OR = 1.23, 95% CI [1.03, 1.48], *p* = 0.020), and environmental concern (OR = 1.21, 95% CI [1.03, 1.42], *p* = 0.018). Variables negatively associated with willingness included perceived non-halal status of insects (OR = 0.15, 95% CI [0.09, 0.25], *p* < 0.001), disgust sensitivity (OR = 0.24, 95% CI [0.17, 0.34], *p* < 0.001), and health risk perception (OR = 0.56, 95% CI [0.43, 0.73], *p* < 0.001). Importantly, unlike for men, social influence was not a statistically significant predictor among women (OR = 1.08, 95% CI [0.85, 1.37], *p* = 0.528).

### 3.6. Application-Oriented Integration Model

**Figure 2:**
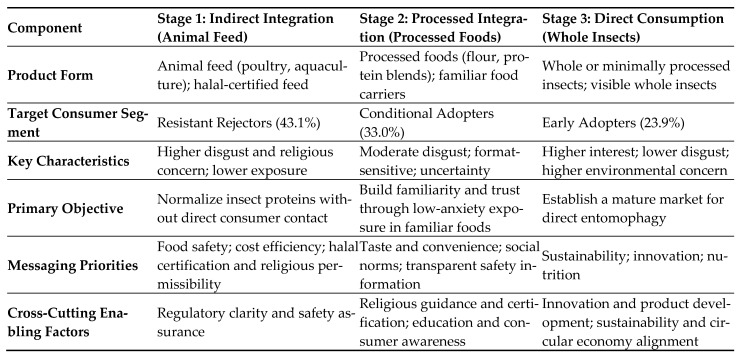
Behavioral pathway for edible insect integration in Saudi Arabia.

The proposed integration model (Figure 2) is grounded in the segmentation results and proposed as a practical framework for future implementation. It translates statistically derived behavioral patterns into application-oriented strategies, while acknowledging that the staged transition hypothesis that consumers will progress across stages over time requires longitudinal empirical validation beyond what the present cross-sectional design can provide.

#### 3.6.1. Segment-Based Market Entry Strategies

Table 5 presents proposed segment-based market entry strategies derived from the observed behavioral patterns.

#### 3.6.2. Multi-Stage Food System Integration Pathway

Stage 1: Indirect Integration (Target: Resistant Rejectors)

Insect-based animal feed (poultry, aquaculture) may represent a feasible entry point. Emphasis may be placed on sustainability messaging, cost savings, and halal certification from religious authorities. This stage may facilitate initial exposure without direct consumer contact.

Stage 2: Processed Food Integration (Target: Conditional Adopters)

Incorporation into familiar foods (flatbreads, labneh, spice mixes, pasta). May reduce perceptual barriers. Strategies may include minimizing visual cues through non-visible ingredients and providing transparent safety labeling. This stage may support familiarity and trust through gradual exposure.

Stage 3: Direct Consumption (Target: Early Adopters)

Whole or visible insect products (roasted locusts, insect snacks). May be more acceptable within this segment. Targeting niche markets and framing products as a revival of traditional ecological knowledge may enhance engagement. This stage may contribute to the development of a more established market for direct entomophagy.

#### 3.6.3. Industry and Policy Implications

For Industry: Product format innovation may be prioritized (e.g., invisible ingredients, familiar carriers). Segmentation-based approaches may be used to guide market entry sequencing (feed → processed → whole). Early adopters may serve as potential market catalysts. Gender-informed marketing strategies may be considered (e.g., sustainability/social for men; safety/trust for women).

For Policymakers: Religious ambiguity may be addressed through official and transparent halal clarification mechanisms. Insect-fed livestock products may be promoted as an acceptable entry point. Edible insects may be integrated into national sustainability and food security strategies (Vision 2030). Support for pilot-scale black soldier fly larvae farming integrated with municipal waste management may be beneficial.

For Food System Development: Insect adoption may be aligned with circular economy principles (waste-to-protein). Edible insects may be positioned as a gradual transition rather than an abrupt dietary change. Further research on safety, allergenicity, and consumer education may be supported.

## 4. Discussion

This study contributes to the literature on sustainable food transitions by linking consumer segmentation to protein transition pathways within circular production systems. The findings suggest that edible insect adoption is not a uniform process but rather a staged transition influenced by psychological, cultural, and experiential factors [7,8,9]. The three key findings of this study are discussed in turn: (1) the identification of three behaviorally distinct consumer segments; (2) the gender-conditioned pathways to acceptance; and (3) the strategic role of indirect consumption as a transitional mechanism.

A key contribution of this study is the identification of indirect consumption pathways as a potentially effective entry strategy. Unlike direct consumption, which faces strong resistance particularly among women, indirect pathways such as insect-based animal feed provide a culturally acceptable starting point for integration. Consistent with the gateway hypothesis [10,24], the present findings suggest that introducing insect proteins first through animal feed may contribute to the normalization of insect-derived products, reduce perceived disgust, and increase familiarity before direct human consumption is attempted. It should be noted that, given the cross-sectional design of this study, the directionality of the relationship between feed acceptance and direct willingness cannot be established causally; longitudinal or experimental designs would be needed to confirm this pathway.

The three identified segments (Resistant Rejectors, Conditional Adopters, and Early Adopters) highlight the importance of accounting for behavioral heterogeneity in intervention design, consistent with calls in the broader literature for segmentation-based approaches to sustainable food transitions [9,10]. The gender-stratified regression results provide the predictor-level mechanism underlying these profiles: the strong positive association between prior experience and willingness (OR = 3.07 for women, OR = 1.95 for men) helps explain why Early Adopters who report the highest rates of prior exposure show markedly higher willingness than Resistant Rejectors, while the strong negative association of religious concern (OR = 0.15 for women) directly maps onto the high religious constraint z-scores observed in the Resistant Rejectors cluster. Together, the two analytical approaches are mutually reinforcing: segmentation identifies who the consumer groups are, and regression identifies why their willingness differs. Resistant Rejectors, who constitute the largest segment (43.1%) and are disproportionately composed of women, may benefit from indirect exposure and religious engagement before direct product targeting is feasible. Conditional Adopters (33.0%) respond to product reformulation and social proof, indicating that the format and framing of insect-based products may be more influential than the source ingredient itself for this group [8]. Early Adopters (23.9%), disproportionately composed of men, may be targeted directly with sustainability and innovation messaging [7].

The gender-conditioned pathways identified here align with the broader literature on gendered food choices yet reveal patterns specific to the Gulf cultural context. The disproportionately strong effect of religious concern among women (OR = 0.15) compared to men (OR = 0.32) warrants further theoretical consideration. One possible interpretation is that women in conservative contexts often serve as primary custodians of household halal compliance, making them more sensitive to perceived religious ambiguity surrounding novel foods. The substantially stronger negative association between perceived non-halal status and willingness among women suggests that religious concerns may reflect uncertainty regarding permissibility rather than outright rejection. Consequently, clear guidance from religious authorities and trusted institutional communication channels may play an important role in reducing misconceptions and facilitating informed consumer decisions regarding edible insects.

This interpretation is further supported by the parallel finding that prior experience is more strongly associated with willingness among women (OR = 3.07) than men (OR = 1.95), which may indicate that direct sensory and social exposure may be more effective than abstract argumentation in shifting attitudes among female consumers, although the cross-sectional design does not permit causal conclusions about this relationship. These findings are also consistent with gender schema and household-role perspectives, which propose that women often assume greater responsibility for household food safety, dietary compliance, and family food choices, thereby increasing sensitivity to perceived food-related risks and religious uncertainty. Because women in Saudi households frequently act as primary food gatekeepers, their attitudes are likely to exert a disproportionate influence on household-level purchase intentions and on the dietary socialization of children, which magnifies the practical importance of addressing the religious knowledge gap among women specifically.

A plausible explanation for these divergent patterns is that men in this sample appear more responsive to novelty and to external social cues consistent with their stronger associations with interest and social influence, whereas women’s willingness is more contingent on direct, trusted experience and on the resolution of safety and religious-related uncertainty. In motivational terms, men’s decisions appear to be driven more by approaching motivation toward dietary innovation, while women’s decisions are more strongly gated by avoidance of perceived health and permissibility risks.

These findings map onto the theoretical frameworks outlined in the Introduction: the dominance of disgust and religious-permissibility barriers reflects the disgust and food-neophobia mechanisms, whereas the gendered weighting of social influence (a significant predictor for men only) is consistent with the subjective-norm component of the Theory of Planned Behavior operating differently across groups.

Comparison with global research reveals both consistency and unique patterns. Saudi men’s acceptance rate (23.0%) is broadly comparable to reported rates in Germany (approximately 23.5%) [7] and Italy (approximately 28.1%) [25], suggesting that male openness to dietary innovation is relatively consistent across cultural contexts. However, Saudi women’s acceptance rate (7.8%) appears lower than that typically observed among European women (18–22%) [7,26], underscoring the additional barrier of religious concerns specific to the Saudi context. Critically, the magnitude of the religious barrier observed among women (OR = 0.15) has not been widely documented in Western or Asian entomophagy studies, which supports the need for culturally adapted and religiously sensitive communication strategies in Saudi Arabia and other Muslim-majority contexts.

Beyond the gender-specific rates discussed above, the overall willingness profile observed in this study aligns with the broader entomophagy literature, in which disgust and food neophobia consistently emerge as the dominant proximal barriers, while environmental motivation and prior exposure act as the principal facilitators [7,8,9]. Our segment structure mirrors the consumer typologies reported by Verbeke [9] and Sogari et al. [10] in Western populations, in which a sizeable rejecting majority coexists with a smaller, environmentally motivated adopter minority. However, the present data diverge from these Western studies in two respects: first, the rejecting segment is proportionally larger (43.1%) than the 20–35% rejection commonly reported in Europe [7]; and second, religious permissibility emerges as a distinct, quantitatively strong barrier that is largely absent from Western segmentation frameworks. This pattern is consistent with the limited Saudi evidence available to date, which likewise reported low baseline acceptance coupled with pronounced cultural sensitivity [27], and extends it by quantifying the gendered magnitude of the religious barrier.

From a broader food system perspective, the findings support the potential value of starting with insect-based animal feed (Stage 1 in our integration model) before targeting direct human consumption. Saudi Arabia’s existing regulatory infrastructure for animal feed and its sizable poultry and aquaculture sectors may offer a feasible entry point [4,5]. Black soldier fly larvae (BSFL) production can be coupled with municipal organic waste streams in a circular economy framework consistent with the Kingdom’s sustainability commitments [28]. BSFL has an established halal status for use in livestock and aquaculture feed [24,29], which further supports its potential viability as a first-entry product in a religiously sensitive market. It is worth noting that the finding that 23.5% of women, compared to only 5.7% of men, believe insects are not halal likely reflects a knowledge gap rather than solely a difference in values. Islamic jurisprudence does not present a uniform consensus on the permissibility of consuming insects beyond locusts, as interpretations vary across scholarly perspectives [27].

Given that locusts are generally recognized as halal within the Saudi context, the observed level of uncertainty among women suggests that this barrier may be addressed through improved awareness and clarification of existing religious perspectives, rather than attempts to alter underlying values.

The regulatory environment is likely to shape how these behavioral pathways translate into market reality, and it differs markedly across jurisdictions. In the European Union, edible insects are classified as novel foods under Regulation (EU) 2015/2283 and require case by-case premarket authorization following a European Food Safety Authority (EFSA) safety assessment; to date only a small number of species (Tenebrio molitor, Locusta migratoria, Acheta domesticus, and Alphitobius diaperinus) have been approved [30]. By contrast, the United States Food and Drug Administration (FDA) adopts a more permissive stance, permitting insects intended for human consumption provided they are produced under good manufacturing and food-safety practices, without a dedicated novel food authorization step [31]. Saudi Arabia occupies an intermediate position: the Saudi Food and Drug Authority (SFDA) regulate novel foods through its Technical Regulation for General Requirements of Novel Foods (SFDA.FD 5013) [32] and an associated approval pathway, while also engaging in international standard-setting through the Codex Alimentarius Commission and the Gulf Standardization Organization. For Saudi Arabia, this means that the staged, feed-first pathway proposed here can advance within an existing and increasingly defined regulatory framework, provided that halal status and food-safety dossiers are clarified early in the approval process.

### Limitations

Several limitations of this study should be acknowledged. First, the cross-sectional design limits causal inference; longitudinal studies tracking segment mobility over time are needed. Second, convenience sampling via social media may over-represent younger, urban, educated adults while under-representing older populations and rural communities. Comparison with national population statistics from the General Authority for Statistics of Saudi Arabia [27] indicates that the present sample over-represents university-educated adults (87.5% vs. approximately 34.5% nationally) and the 18–34 age group (42.5% vs. approximately 36.2% nationally), while under-representing adults aged 51 years and above. Regional representation also differs modestly from national distributions, including the northern region (5.9% of the sample vs. approximately 4.8% nationally). These deviations likely reflect the online recruitment strategy and Saudi Arabia’s high internet penetration and digital engagement, particularly among younger and more educated populations [33]. Consequently, the findings are most applicable to urban, educated, and digitally connected Saudi adults; generalization to older or less digitally engaged populations should be made with caution. Third, the gender imbalance in the sample (65.7% women vs. 34.3% men) reflects typical online survey response patterns. Fourth, social influence was assessed using a single-item measure, which limits psychometric robustness. Fifth, religious perception was operationalized through a single dichotomous belief statement; this simplification does not capture the nuanced range of Islamic jurisprudential opinions. Sixth, willingness to consume was assessed as a behavioral intention rather than actual consumption behavior. Seventh, all data were self-reported and therefore subject to social desirability bias. Finally, the proposed integration model has not yet been empirically tested through pilot interventions.

## 5. Conclusions

In addressing the study’s guiding questions, three conclusions emerge: edible-insect adoption in Saudi Arabia is segmented rather than uniform; acceptance follows gender-conditioned pathways; and indirect consumption offers a culturally pragmatic transition mechanism. The study suggests that behavioral segmentation provides a powerful framework for understanding and facilitating the adoption of edible insects in culturally sensitive contexts. Three distinct segments—Resistant Rejectors, Conditional Adopters, and Early Adopters—exhibit different barriers, motivators, and readiness levels. Gender-specific pathways are evident, with men more responsive to social and environmental framing, and women’s adoption more strongly associated with prior experience, safety perceptions, and religious interpretation.

By linking consumer profiles to targeted application strategies, the proposed model offers a potential roadmap for integrating alternative proteins into sustainable food systems, particularly among urban, educated, and digitally connected Saudi adults who constitute the most accessible early market. The staged pathway from animal feed to processed foods to direct consumption may provide a culturally acceptable transition mechanism that respects consumer heterogeneity while advancing food security objectives aligned with Saudi Vision 2030. These conclusions should be extended to older and rural populations with caution, as these groups were under-represented in the present sample and may exhibit meaningfully different acceptance profiles.

The present study has several notable strengths that enhance the robustness and relevance of its findings. First, it is based on a large national sample (*n* = 2208), providing sufficient statistical power for subgroups and gender-stratified analyses. Second, the study adopts an integrated analytical approach by combining behavioral segmentation with ordinal logistic regression, allowing for both the identification of consumer groups and the examination of the underlying predictors of willingness. Third, the study explicitly incorporates cultural and religious dimensions into the analytical framework, which are often underrepresented in entomophagy research, particularly in non-Western contexts. Fourth, the findings extend beyond descriptive analysis by translating behavioral patterns into a structured, application-oriented integration model, offering practical insights for industry and policy. Finally, the identification of indirect consumption (insect-based animal feed) as a feasible entry point provides empirically grounded support for a staged transition pathway in culturally sensitive contexts.

### Future Research

Future research should: (1) validate the proposed segmentation framework in other Muslim-majority contexts; (2) conduct segment-stratified analyses to examine whether key predictors identified here (e.g., disgust sensitivity, religious perceptions, and prior experience) operate differently across consumer segments, thereby advancing a more integrated behavioral model; (3) integrate the behavioral framework with the nutritional and environmental characterization of regionally relevant insect species (e.g., locusts, black soldier fly larvae); (4) employ experimental or quasi-experimental designs to test whether segment-targeted interventions can produce sustained changes in attitudes over time; and (5) further examine the role of religious guidance and institutional communication in shaping insect acceptance, particularly in addressing the observed knowledge gap regarding halal status among women.

## Figures and Tables

**Table 1:** Socio-demographic characteristics of the study participants (*n* = 2208).

Variables	Men, 758 (34.3%)	Women, 1450 (65.7%)	Test Statistic	df	*p*-Value
Age(years)			χ^2^ = 27.45	2	0.001 ***
18–34	289 (38.1%)	650 (44.8%)			
35–50	250 (33.0%)	324 (22.3%)			
≥51	219 (28.9%)	476 (32.8%)			
Education level			χ^2^ = 1.87	1	0.171
≥University	641 (84.5%)	1291 (89.0%)			
≤Secondary school	117 (15.5%)	159 (11.0%)			
Employment status			χ^2^ = 1.62	1	0.203
Employed	631 (83.2%)	1223 (84.3%)			
Not employed	127 (16.8%)	227 (15.7%)			
Monthly income (SAR)			χ^2^ = 0.83	1	0.362
>10,000 SAR	541 (91.9%)	1048 (93.4%)			
≤10,000 SAR	48 (8.1%)	74 (6.6%)			
Region of residence			χ^2^ = 139.15	4	0.001 ***
Eastern	280 (39.9%)	421 (60.1%)			
Northern	65 (49.6%)	66 (50.4%)			
Western	110 (39.7%)	167 (60.3%)			
Southern	140 (37.5%)	233 (62.5%)			
Central	163 (22.5%)	563 (77.5%)			

Note: Values are presented as *n* (%). Chi-square tests were used to assess gender differences. *** *p* < 0.001.

**Table 2:** Cluster centroids (standardized z-scores) and cluster sizes for the three behavioral segments.

Variable (z-Score)	Resistant Rejectors	Conditional Adopters	Early Adopters
Disgust (EAQ-D)	+0.87	+0.12	−1.24
Interest (EAQ-I)	−0.91	+0.08	+1.31
Environmental concern	−0.52	+0.21	+1.02
Prior experience	−0.78	+0.15	+1.45
Social influence	−0.63	+0.34	+0.98
Religious constraints	+0.95	+0.18	−1.12
Cluster size, *n* (%)	951 (43.1%)	729 (33.0%)	528 (23.9%)
Women, *n* (%) ^a^	685 (72.0%)	423 (58.0%)	216 (41.0%)
Men, *n* (%) ^a^	266 (28.0%)	306 (42.0%)	312 (59.0%)

Note: Values are standardized z-scores (mean = 0, SD = 1). Positive values indicate above-average levels. All variables differed significantly across clusters (one-way ANOVA, all *p* < 0.001). ^a^ Gender distribution differed significantly across clusters (χ^2^(2) = 136.72, *p* < 0.001).

**Table 3:** Gender differences in behavioral determinants of edible insect consumption in Saudi adults.

Determinant	Men (*n* = 758)	Women (*n* = 1450)	Test Statistic	df	*p*-Value	Effect Size
Environmental concern (mean ± SD)	4.20 ± 1.17	3.32 ± 1.34	t(2206) = 15.42	2206	<0.001	d = 0.69
Health risk beliefs (mean ± SD)	2.05 ± 1.21	3.20 ± 1.32	t(2206) = 20.14	2206	<0.001	d = 0.90
Prior consumption experience (% yes)	24.1%	7.8%	χ^2^(1) = 115.73	1	<0.001	V = 0.27
Social influence (% agreeing)	32.2%	15.3%	χ^2^(1) = 89.46	1	<0.001	V = 0.24
Religious belief (insects not halal, % yes)	5.7%	23.5%	χ^2^(1) = 121.18	1	<0.001	V = 0.35
Willing to consume insects (% willing)	23.0%	7.8%	χ^2^(1) = 106.84	1	<0.001	V = 0.28

Note: Values are presented as mean ± standard deviation or percentages (%). Independent-samples *t*-tests were used for continuous variables, and chi-square tests were used for categorical variables. Effect sizes are reported as Cohen’s d for continuous variables and Cramér’s V for categorical variables.

**Table 4:** Feed acceptance (EAQ-F) and direct willingness to consume edible insects by segment and gender.

Segment	Men (EAQ-F)	Women (EAQ-F)	Men (Direct Willingness)	Women (Direct Willingness)
Resistant Rejectors	3.2 ± 1.4	2.8 ± 1.2	2.5 ± 1.1	2.3 ± 1.0
Conditional Adopters	4.1 ± 1.5	3.6 ± 1.3	3.5 ± 1.2	3.0 ± 1.1
Early Adopters	5.2 ± 1.6	4.8 ± 1.5	4.5 ± 1.4	4.5 ± 1.3

Note: Values are presented as mean ± standard deviation. Feed acceptance (EAQ-F) was measured on a 1–7 scale, while direct willingness to consume edible insects was measured on a 1–5 scale; comparisons are therefore descriptive and should be interpreted cautiously.

**Table 5:** Feed acceptance (EAQ-F) and direct willingness to consume edible insects by segment and gender. Segment-based market entry strategies for promoting edible insect adoption in Saudi Arabia.

Segment	Dominant Barrier	Suggested Entry Strategy	Product Form	Messaging Focus
Resistant Rejectors	Disgust + religious concern	Indirect exposure	Animal feed (poultry, fish)	Safety, religious permissibility
Conditional Adopters	Uncertainty	Product reformulation	Processed foods (flour, protein blends)	Taste, convenience, social proof
Early Adopters	Low barrier	Direct targeting	Whole or minimally processed insects	Sustainability, innovation, nutrition

Note: Values are presented as mean ± standard deviation. Feed acceptance (EAQ-F) was measured on a 1–7 scale, while direct willingness to consume edible insects was measured on a 1–5 scale; comparisons are therefore descriptive and should be interpreted cautiously.

## Data Availability

The data sets used and analyzed during the current study are available from the corresponding authors on reasonable requests.

## Abbreviations

The following abbreviations are used in this manuscript:

EAQ	Entomophagy Attitude Questionnaire
EAQ-D	Disgust Subscale
EAQ-I	Interest subscale
EAQ-F	Feeding Animals subscale
